# Microfluidic deep mutational scanning of the human executioner caspases reveals differences in structure and regulation

**DOI:** 10.1038/s41420-021-00799-0

**Published:** 2022-01-10

**Authors:** Hridindu Roychowdhury, Philip A. Romero

**Affiliations:** 1 Department of Biochemistry, University of Wisconsin-Madison, Madison, WI USA; 2 Department of Chemical & Biological Engineering, University of Wisconsin-Madison, Madison, WI USA; 3 The University of Wisconsin Carbone Cancer Center, Madison, WI USA

**Keywords:** Proteases, Enzyme mechanisms, Biotechnology, Screening

## Abstract

The human caspase family comprises 12 cysteine proteases that are centrally involved in cell death and inflammation responses. The members of this family have conserved sequences and structures, highly similar enzymatic activities and substrate preferences, and overlapping physiological roles. In this paper, we present a deep mutational scan of the executioner caspases CASP3 and CASP7 to dissect differences in their structure, function, and regulation. Our approach leverages high-throughput microfluidic screening to analyze hundreds of thousands of caspase variants in tightly controlled in vitro reactions. The resulting data provides a large-scale and unbiased view of the impact of amino acid substitutions on the proteolytic activity of CASP3 and CASP7. We use this data to pinpoint key functional differences between CASP3 and CASP7, including a secondary internal cleavage site, CASP7 Q196 that is not present in CASP3. Our results will open avenues for inquiry in caspase function and regulation that could potentially inform the development of future caspase-specific therapeutics.

## Introduction

Caspases are a ubiquitous family of cysteine proteases that play fundamental roles in programmed cell death and inflammation [1]. These enzymes have numerous ancillary roles in organismal development and homeostasis including cell differentiation, synaptic pruning, and cytokine processing [2, 3]. In humans, there are 12 expressed members of the family, with Caspase-3, −6, −7, −8, and −9 primarily involved in apoptosis, and the others involved in pyroptosis and inflammation [1]. All caspases have a conserved core proteolytic domain and a variable N-terminal domain that is involved with regulation of enzyme activity [3].

Dysregulation of caspase activity is associated with cancer, neurodegeneration, vascular ischemia, and inflammatory diseases [1, 4, 5]. Consequently, these enzymes represent important therapeutic targets to treat a variety of human diseases [4]. However, despite their central role in human biology and disease, every caspase-targeting drug candidate has failed to pass through clinical trials [6, 7]. A key challenge for therapeutic development has been the caspase family’s highly conserved proteolytic domain, which makes it difficult to selectively target one particular member and leads to off-target effects [8]. A deeper understanding of caspase structure, function, and regulation may eventually lead to small molecule modulators that selectively target members of the caspase family and open the door for novel therapeutics [6, 9, 10].

In this work, we develop a high-throughput microfluidic platform for caspase screening and apply it to systematically map sequence-function relationships in the human executioner caspases [11, 12]. Our microfluidic system consists of a fully integrated lab-on-a-chip that combines the addition of a fluorogenic substrate, incubation of the enzyme reaction, and fluorescence measurement. Our microfluidic chip can perform kinetics-based screening on millions of caspase variants. We applied our screening system to perform deep mutational scanning (DMS) on caspase-3 (CASP3) and caspase-7 (CASP7). The DMS data displayed known and expected signatures of caspase structure and function, but also revealed important differences between CASP3 and CASP7 that may be related to allosteric regulation and protein stability. Future exploration of the differences between human caspases may lead to more targeted drug design efforts.

## Results

### A microfluidic platform for ultra-high-throughput screening of caspases

High-throughput screening is an important tool for studying protein structure and function [13]. Caspases are challenging to screen because their activity cannot be readily linked to cell growth or cellular fluorescence. Furthermore, caspases’ high catalytic rates make any cell-based assay difficult because the proteolytic cleavage reactions occur on significantly faster timescales than cell growth or fluorescent protein production. We developed a droplet microfluidic platform capable of in vitro, kinetics-based screening of millions of caspase variants.

**Fig. 1:**
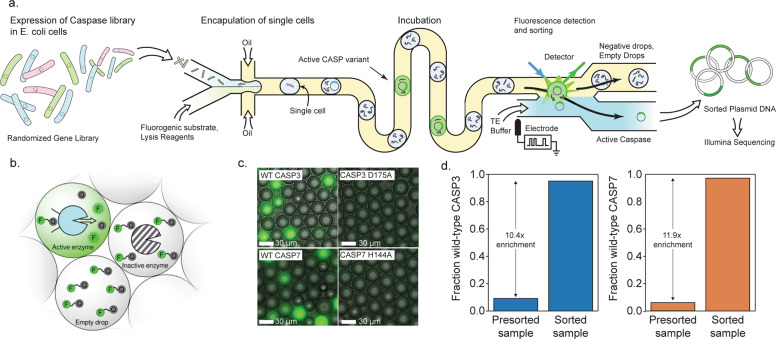
A droplet microfluidic platform for screening caspases. **a** A schematic of our microfluidic screening system. A dilute suspension of *E. coli* expressing caspase variants are injected into a microfluidic device and individual cells are encapsulated into microdroplets containing lysis reagents and a fluorogenic caspase substrate. The cells are lysed, the enzyme reaction is incubated on-chip, and the fluorescence of each droplet is analyzed using a laser. The fluorescent droplets are then sorted by electrocoalescence with an aqueous stream that collects the sorted plasmids for downstream analysis. **b** Droplets containing active caspase variants will fluoresce, whereas empty droplets and droplets containing inactive caspases will not. **c** Microscopy images of droplets containing active WT CASP3 and WT CASP7 display strong green fluorescence, while droplets containing the inactive CASP3 D175A and CASP7 H144A variants remain dark. **d** Results of a mock screen demonstrate over tenfold enrichment of active CASP3 and CASP7.

Our microfluidic system encapsulates single *E. coli* cells, each expressing a unique caspase variant, into ~10 picoliter microdroplets that contain cell lysis reagents and a fluorogenic peptide substrate (Fig. 1a). The droplets physically separate each cell and allow enzyme reactions to proceed in isolation. After encapsulation, the cells quickly lyse, releasing the expressed caspase and allowing it to interact with the substrate. The droplets are then incubated in an on-chip continuous flow reactor for ~3 min to allow the reaction to proceed. We found these short incubation times were necessary to separate highly active caspases from variants with severely attenuated activity. After incubation, each droplet is scanned with a laser fluorimeter and droplets displaying high fluorescence signals are sorted for downstream analysis. Our microfluidic platform is capable of screening 360,000 caspase variants per hour, while consuming only ~100 μL of assay reagents.

We tested the ability of our emulsion-based assay to distinguish active CASP3 from an inactive D175A mutant (Fig. 1c, d). We encapsulated cells expressing each variant and analyzed the droplets using fluorescence microscopy. Droplets that contained active CASP3 displayed a strong fluorescence signal, while droplets with the inactive mutant had no measurable fluorescence. We next tested our assay on-chip using the integrated laser fluorimeter. The active enzyme was easily distinguished from the inactive mutant, with the average CASP3 droplet signal being at least fivefold greater than the inactive mutant (Supplementary Fig. 1a, b).

We next evaluated our microfluidic system’s ability to enrich active caspases from a mixed variant population. We performed a mock sorting experiment by combining active CASP3 with a tenfold excess of an inactive empty plasmid control. We ran this mixed control population through our microfluidic system and sorted droplets with high fluorescence values. We then analyzed the proportion of active CASP3 versus empty plasmid by agarose gel electrophoresis (Supplementary Fig. 1c). We found the initial population contained 9% active CASP3, as expected, and the sorted population contained 95% active CASP3 (Fig. 1d). These results indicate that our system can enrich active caspases by at least tenfold, which is ample for high-throughput screening.

### Deep mutational scanning of the human executioner caspases

Caspases 3, 6, and 7 are referred to as the executioner caspases because they perform the large-scale cellular proteolysis that leads to apoptosis. These enzymes share similar in vitro substrate preference, however have been implicated in nonredundant cellular roles that cannot be fully explained by either structural differences or protein expression levels [14–16]. It is likely that subtle differences in their primary amino acid sequence may explain their in vivo and in vitro functional profiles.

We leveraged our microfluidic screening platform to systematically map sequence-function relationships for CASP3 and CASP7. We generated CASP3 and CASP7 libraries using error-prone PCR. These libraries contained 2–4 amino acid substitutions per variant and ~25% of these variants were active caspases. We screened these CASP3 and CASP7 libraries for active caspases using our microfluidic platform. We screened each library in triplicate to evaluate the reproducibility of our methods and to ensure the robustness of our results. For each screening run, we analyzed over 1.5 million caspase variants on average and sorted 4 × 10^5^–7 × 10^5^ active variants for downstream DNA sequencing analysis (Supplementary Table 1).

**Fig. 2:**
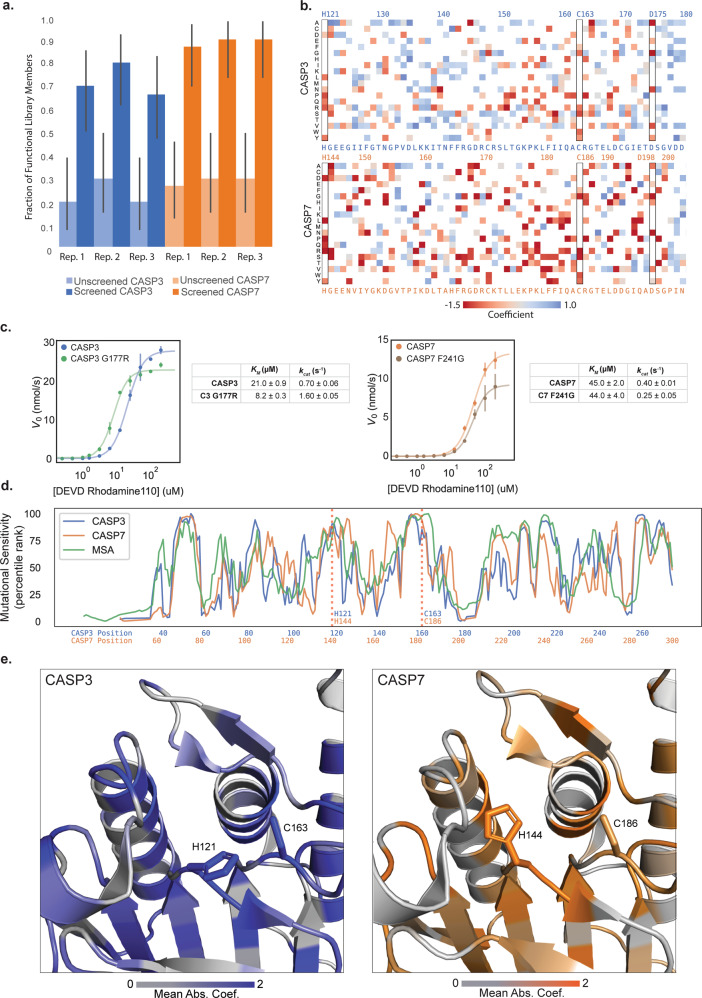
Deep mutational scanning of CASP3 and CASP7. **a** Enrichment of active caspase variants in screened libraries. The fraction of active caspase variants was measured in a plate-based assay before and after screening. The error bars represent the 95% binomial proportion confidence intervals. All replicates showed significant enrichment. **b** A heatmap of mutational coefficients surrounding the active site of CASP3 and CASP7. Mutations that are red have negative coefficients, corresponding to deleterious mutations. Mutations that are blue are positive and are either neutral or activating mutations. White boxes are mutations that did not appear on our DMS analysis. The outlined columns highlight the active site histidine and cysteine residues, as well as the internal aspartate where zymogen maturation occurs. **c** Kinetic analysis of putatively activating mutations, CASP3 G177R and CASP7 F241G. Kinetic parameters were estimated from the Hill equation. **d** The mutational tolerance of CASP3, CASP7, and the caspase family multiple sequence alignment (MSA) across sequence positions. The mutational tolerance at each position was calculated as the mean absolute value of all mutation coefficients at that position and plotted as a percentile rank. **e** The three-dimensional structures of the CASP3 and CASP7 active sites with their mutational tolerance scores mapped onto the structure. The active site residues are labeled and are strongly colored, indicating low tolerance to mutation.

We verified the sorted caspase variants were active enzymes by retransforming the genes into *E. coli* and assaying individual clones in a plate-based format. The initial unsorted libraries were 20–25% functional, while the sorted libraries were 60–90% functional, indicating strong enrichment of functional sequences (Fig. 2a). We then sequenced all six sorted samples and their corresponding initial unsorted libraries using Illumina sequencing. The data displayed excellent reproducibility across the three experimental replicates for CASP7 and two experimental replicates for CASP3 (Supplementary Fig. 2). The third CASP3 replicate displayed poor agreement with the first two replicates (Supplementary Fig. 2), and was excluded from further analysis. The third CASP3 replicate had significant false positive sequences as indicated by its high fraction of inactive sequences (Fig. 2a).

We used our DMS data to build large-scale maps describing how individual mutations affect CASP3 and CASP7 activity (Fig. 2b). These maps display expected mutational patterns for both caspases. Substitution of the active site cysteine and histidine residues is highly deleterious. Mutations to large aromatic residues in the hydrophobic core are not tolerated, whereas polar substitutions on the surface of the protein and chemically conservative mutations are generally more lenient. The internal processing sites D175 in CASP3 and D198 in CASP7, which are essential for maturation of zymogenic caspases to mature proteases, are also intolerant to mutation.

In addition to corroborating known and expected mutational patterns, our data also revealed new mutations that appear to enhance caspase activity. Our DMS analysis identified G177R as an activating mutation in CASP3. To validate this finding, we constructed, expressed, and measured CASP3 G177R’s enzyme kinetic properties (Fig. 2c). CASP3 G177R’s catalytic efficiency (*k*_*cat*_*/K*_*M*_) is over two-fold higher than wild-type CASP3. G177 is distant from CASP3’s active site, but is located adjacent to the internal zymogen processing site D175. Mutations at position 177 could enhance enzyme activity through allosteric or conformational rearrangements related to CASP3’s native activation mechanisms. CASP7 F241G was another putative activating mutation identified from our DMS analysis. Further kinetic characterization of this mutant found it was an active caspase, but actually had a lower turnover number (*k*_*cat*_) relative to wild-type CASP7. This discrepancy between the bulk assay and the droplet screen could be the result of enzyme expression level, since our droplet assay considers total activity that is not normalized to enzyme concentration. All enzyme kinetic measurements are summarized in Supplementary Table 2.

We aggregated the individual mutational effects to obtain the mutational tolerance of each position in CASP3 and CASP7’s primary sequence. This mutational tolerance is related to a site’s importance for caspase function and allows us to analyze broader sequence and structural features. The site-wise mutational tolerance profiles of CASP3 and CASP7 are generally very similar, and also agree closely with profiles generated from a multiple sequence alignment (MSA) of natural caspases (Fig. 2d). The beta-sheets that comprise the proteins’ core are less mutable than the exterior helices, and the active site is evolutionarily conserved in the MSA and was also seen to be immutable in our deep mutational scan (Fig. 2e).

### Contrasting mutational profiles reveals functional differences across executioner caspases

Humans possess 12 separate caspases that all diverged from a common ancestor and share the same structurally conserved proteolytic domain. Despite their highly similar structure and biochemical activity, each caspase’s regulation and cellular targets are unique and confer numerous non-redundant physiological roles. We explored our CASP3 and CASP7 sequence-function profiles to better understand functional differences between highly similar members of the caspase family.

**Fig. 3:**
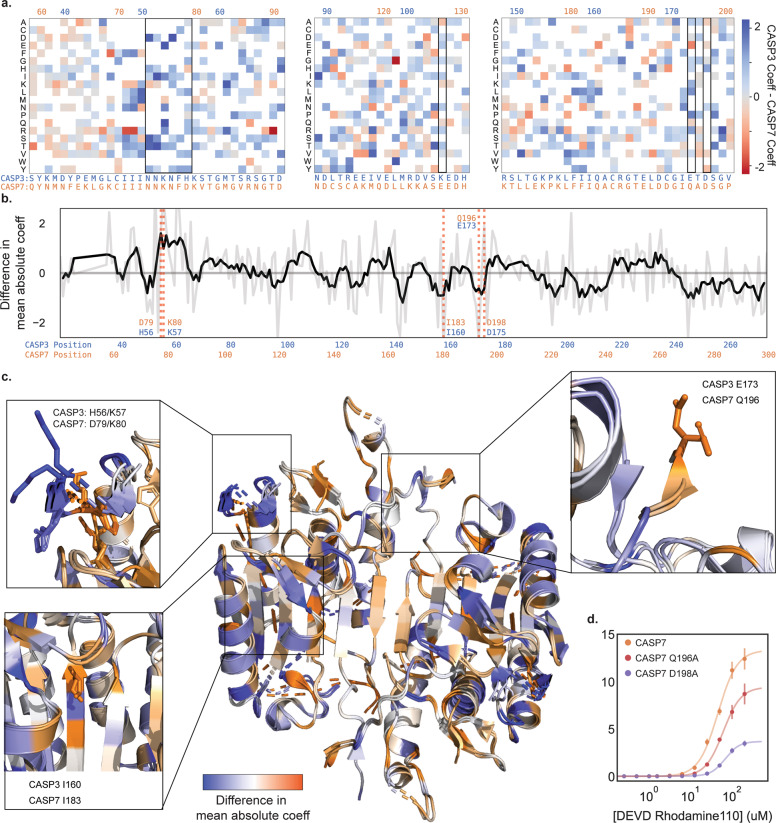
Divergence in the mutational landscapes of Caspase-3 and -7. **a** A heatmap of mutation coefficient differences between CASP3 and CASP7. Blue indicates a larger coefficient in CASP3 and orange indicates CASP7 has a larger coefficient. **b** Differences in mutational tolerance between CASP3 and CASP7. The light gray line shows the difference in a the mean absolute coefficient of a site between CASP3 and CASP7, and black line is a moving average to highlight general differences. Positive values are positions where CASP3 has a larger mean absolute coefficient and thus mutations at that site have a larger effect. Negative values are where CASP7 has a larger effect. The red dotted lines indicate positions of interest. **c** A mapping of the difference in mean absolute coefficient onto the aligned CASP3 and CASP7 structures (2H5J and 2QL5, respectively). The expanded boxes highlight the sequence regions shown in (**a** and **b**). **d** Kinetic analysis of CASP7 Q196A and CASP7 D198A.

We compared the mutational profiles of CASP3 and CASP7 to identify sites that display differing mutational tolerance and may have functionally diverged during caspase evolution and specialization (Fig. 3a, b). One notable sequence position was E173 in CASP3 and the equivalent residue Q196 in CASP7 (Fig. 3c). CASP3 can tolerate any substitution at this position, whereas CASP7 can only accept substitution to glutamic acid. We verified this finding by performing enzyme kinetics measurements on CASP7 Q196A (Fig. 3d). We found CASP7 Q196A had significantly diminished catalytic efficiency relative to wild-type CASP7. Intriguingly, Q196 is a known important regulatory site in CASP7 that is cleaved by Cathepsin G to activate procaspase-7 [17]. While Cathepsin G is not present in our *E. coli*-based screen, it is possible that CASP7 can self-activate at this site and amino acid substitutions at this site reduce the pool of active enzyme.

Another differing sequence region were the adjacent sites H56/K57 in CASP3 and the corresponding D79/K80 in CASP7 (Fig. 3c). Our analysis indicates that CASP3 residues H56 and K57 are completely insensitive to mutation, except for H56P. In CASP7, D79 and K80 show complete mutational intolerance, with all substitutions being deleterious. These residues are located in a solvent exposed loop that displays identical conformations between CASP3 and CASP7 and is located near the substrate binding site. Inspection of the crystal structures reveal that CASP7 has an extensive salt-bridge network in this region, while CASP3 does not. Presumably, mutations in CASP7 disrupt the salt-bridge network and lead to an altered conformation or destabilization in the protein structure.

A final site to note was I160 in CASP3 and I183 in CASP7 that are located within a beta-sheet in the core of the enzyme (Fig. 3c). I160T and I160S are well tolerated in CASP3, but CASP7 cannot tolerate any substitutions at I183. The packing environment of I160 and I183 are identical in the crystal structures, with the neighboring sidechains matching with sub-angstrom alignment. It is possible that substitutions at these sites are always destabilizing, but CASP3 has additional stabilization from elsewhere in the protein to permit these destabilizing core mutations.

## Discussion

Caspases play a key role in numerous biological processes that are important for human health and disease. A deeper understanding of caspase structure, function, and regulation could open the door to novel therapeutic approaches [4, 7, 18]. In this work, we performed DMS on CASP3 and CASP7 to reveal differences between highly similar members of the caspase family.

This work was enabled by our high-throughput droplet microfluidic screening platform that analyzes over 300,000 variants per hour in a highly controlled in vitro reaction environment [11, 19]. Our device allowed us to have strict control over how long the proteolytic reactions were allowed to occur, which allowed us to more effectively differentiate caspase variants with altered activity. Cell-based assays that rely on proteolytic reporters and fluorescence-activated cell sorting (FACS) occur on much longer timescales and thus cannot distinguish WT-like activity from variants with severely diminished activity [20]. Even catalytically “dead” active site mutants such as CASP3 D175A display enough enzyme activity to completely hydrolyze the substrate within a few hours [21, 22]. The ability to screen caspases based on fast reaction timescales is necessary to distinguish finer functional differences [23, 24]. While our microfluidic screening platform provided these key advantages, it also presented technical challenges. Compared to standardized experimental platforms such as FACS, the design and optimization of our screen and surrounding workflow required dozens of rounds of optimization and engineering before we could reliably use it screen enzymes [25]. This upfront labor presents a non-trivial roadblock for other researchers looking to adapt the platform, especially if their lab is not already equipped to fabricate and use microfluidic devices [25]. We hope that as more demonstrations of our platform’s utility follow, other researchers or private industries will begin to create easy-to-use prefabricated microfluidic chips and platforms that decrease barriers to entry [20, 26].

We mapped the effects of 1644 amino acid substitutions in CASP3 and 1772 amino acid substitutions in CASP7—roughly one-third of all possible single amino acid substitutions. Our results corroborated findings from previous research, such as mutational intolerance of the catalytic cysteine and histidine residues and other known allosteric and processing sites. We also observed mutational constraints in both enzymes that closely follow our understanding of protein stability from the structural perspective, such as the destabilizing effects of disrupting salt bridges or mutations to the hydrophobic cores.

We additionally characterized the two putatively activating mutations, CASP3 G177R and CASP7 F241G. CASP3 G177 is located two positions downstream of the D175 processing site and exists on an unstructured loop that is not visible in any crystal structure. Mutation to arginine at position 177 significantly lowered the *K*_*m*_ of CASP3, but left the *k*_*cat*_ unchanged. The CASP7 F241G mutation displayed near wild-type activity, with no significant change in *K*_*M*_ or expression, but with decreased *k*_*cat*_. We hypothesize that disrupting core hydrophobic interactions may change the geometry and stability of the enzyme active site and make substrate proteolysis less efficient. These mutations would be difficult to identify without large-scale screening of random mutant libraries [27].

We compared the mutational profiles between CASP3 and CASP7 to identify sites with differing mutational tolerance that may have diverged during caspase evolution and specialization [28, 29]. As expected, a majority of the sites displayed similar mutational tolerance, but a small subset showed statistically significant differences. We identified several key sites that may hold potential for future drug design. CASP7 D79/K80 forms a structurally crucial salt-bridge network that is not observed in CASP3. One may imagine designing a drug that could disrupt that network and selectively inhibit CASP7 while leaving CASP3 function relatively untouched [4, 6, 9, 18].

Our study had several key limitations. First, we chose to express caspases in *E. coli* due to the simplified molecular biology, high transformation efficiency, and the relative insensitivity of bacteria to caspase overexpression. The enzymes expressed in *E. coli* lack glycosylation and must operate in the absence their native regulatory partners such as other caspases and XIAP [3, 30, 31]. In addition, we assayed caspase activity on a single fluorogenic substrate that is likely not fully representative of their diverse cellular targets [32, 33]. These factors could bias our results and reduce the relevance for caspase function in human cells.

Another limitation of DMS studies in general is the inability to dissect detailed molecular mechanisms [34, 35]. Our DMS measurements describe *how* amino acid substitutions affect caspase activity, but they don’t explain *why*. A mutation that decreases caspase activity could be the result of changes in protein expression, stability and folding, catalytic rate constants, substrate specificity, allosteric regulation, and more. Further biochemical characterization of individual mutants is necessary to obtain a complete picture of inner molecular workings of caspases.

Our results have highlighted several interesting future research directions. Residue Q196 appears to play an important role in CASP7 regulation, presumably because it serves as a secondary cleavage site for activation. Previous work found cleavage at the canonical D198 site or Q196 both activate CASP7, however the Q196 isoform is resistant to inhibition by BIR and XIAP [17, 36]. We hypothesize that wild-type CASP7 exists as two different cleavage isoforms and alanine mutation at each of these two processing sites effectively shuts off formation of one of these isoforms. More specifically, the Q196A variant allows us to study the activity of the D198 cleavage isoform in isolation, and the D198A variant allows us to study the Q196 cleavage isoform. Our kinetic analysis of CASP7 Q196A and CASP7 D198A found both variants have diminished activity, but mutation at the canonical processing site D198 attenuates CASP7 activity more than at Q196. This result suggests the two different CASP7 isoforms may have mechanistic differences that account for their differences in kinetics. Considering previous research demonstrated the two isoforms have distinct interactions with native human inhibitory partners [17], it may be that the endogenous *E. coli* serine protease inhibitor Ecotin may also have distinct inhibitory modes with the recombinantly expressed CASP7 isoforms—since all our kinetic analyses were conducted in lysate, those effects could significantly alter the kinetics of the CASP7 mutants.

Further, exploring the possibility of leveraging sites like CASP7 D79/K80 to develop selective caspase inhibitors could be prudent to the field of drug design [6, 37]. Demonstrating practical translational results from our screen could open possibilities for using DMS for targeted and selective drug design for many other peptide targets [4, 38–40]. Developing small molecule modulators that can selectively inhibit or activate members of the caspase family could open the door for novel therapeutics for a wide variety of human diseases [6, 41]. Designing such molecules is incredibly challenging given the highly conserved structures and functions of caspases, and our limited understanding of protein dynamics and regulation [42, 43]. DMS studies could narrow the space of potential target sites by directly and empirically correlating thousands of mutations to their functional effects and finding key protein features that functionally differ in closely related families of proteins [13].

## Materials and methods

### Caspase library generation

Δpro-domain *casp3* and *casp7* genes were amplified using error-prone PCR to introduce random mutations. Error-prone PCR was performed following a protocol calling for 50 uM MnCl_2_ to decrease the fidelity of *Taq* polymerase [44]. We did 15 amplification cycles, introducing ~4.5 nucleotide mutations in the gene. We subsequently purified the amplified product, digested it overnight with DpnI to remove remaining wild-type plasmid inserts, and cloned the insert back into *pET22b* using Circular Polymerase Extension Cloning (CPEC) [45, 46].

The CPEC product was purified and used to transform electrocompetent *E. coli* C43(DE3) cells (Lucigen). Transformed cells were recovered for 45 min at 37 °C then and diluted into 200 mL of sterile LB media with the added carbenicillin. Once the culture’s optical density (OD) approached the lower detection limit of our spectrometer (OD_600_ = 0.2), the culture was concentrated, and freezer stocks of 25% glycerol were made and stored at −80 °C. Each library had roughly 10^7^ transformants. Ten transformants were picked from each library and their plasmids sequenced to find that each library had ~2.5 amino acid substitutions per library member.

### Plate reader-based caspase activity assay

Individual clones from the mutagenized libraries were incubated in Magic Media (Invitrogen) for 18 h at 30 °C. Cells were pelleted and resuspended in solution 50 mM Tris, ph 7.4, 50 mM KCl after decanting the supernatant media to achieve a density of 1 OD_600_/mL. 200 uL of resuspended culture was added to a black 96-well plate. 200 uL of assay reagent (0.3× BugBuster (Invitrogen), 20 uM DEVD-Rhodamine-110 (Bachem), 50 kU/mL Lysozyme, 50 mM Tris pH 7.4, 50 mM KCl, 100 uM EDTA) was added to the plate and the fluorescence (excitation at 480 nm, emission at 530 nm) over time measured on a plate reader. Sequences with >50% of the wild-type activity were considered to be functional.

### Caspase kinetics experiments

*E. coli* C43(DE3) cells expressing WT CASP3, WT CASP7, CASP3 D175A, CASP7 H144A, CASP3 G177R, CASP7 Q196A, or CASP7 D175A were grown for 18 h at 30 °C in Magic Media (Invitrogen). Cells were centrifuged and resuspended in 50 mM Tris, ph 7.4, 50 mM KCl to 1 OD mL. Enzyme concentration was determined using active site titration [47] using the irreversible pan-Caspase inhibitor Z-VAD-FMK (Promega) and observing residual activity upon addition of assay reagents described above. Kinetic parameters were determined by observing proteolytic activity with a titrated range of the DEVD-Rhodamine-110 (Bachem) and fitting the observed initial velocity to the Hill equation [47].

### Microfluidic device fabrication

An initial layer of photoresist resin, SU-8 3010, was coated onto a mirrored silicon wafer (University Wafers) and centrifuged at 1500 *rpm* to achieve 15 um layer height. A photomask (Supplementary Fig. 3) of the first layer of the microfluidic device was placed on the layer and 100 J/cm^2^ of UV light is used to polymerize the features. The wafer was baked at 95 °C for 10 min to catalyze the polymerization. A second 25 um layer of SU8-3025 was coated onto the wafer by spinning at 4000 rpm, and similarly polymerized with the second photomask (Supplementary Fig. 2b) to create the incubation line and baked again. Undeveloped photoresist is washed off with SU-8 developer (1-methoxy-2-propanol acetate, MicroChem).

The wafer was then used to create a relief in un-polymerized poly dimethyl siloxane (PDMS) (Dow Corning Sylgard^®^ 184, 11:1 polymer:cross-linker ratio), which was then polymerized by baking at 75 °C. Inlet and outlet holes are punched with a 0.5 mm biopsy corer. The device was then thoroughly washed with isopropanol and double-deionized water and then plasma treated alongside a clean glass microscope slide, to which it was subsequently bonded. Prior to use, microfluidic channels were filled with Aquapel (Pittsburgh Glass Works) to ensure hydrophobicity, and then baked for 10 min at 100 °C to vaporize any Aquapel left in the channels.

### Microfluidic caspase screening

10 uL of either CASP3 or −7 library glycerol stocks was used to inoculate 5 mL of auto-induction media (Invitrogen Magic Media) and allowed to incubate and express for 18 h at 30 °C. The cultures were pelleted and resuspended in the assay buffer (50 mM Tris pH 7.4, 50 mM KCl, 100 uM EDTA) to a concentration of 0.075 OD_600_ to form the 2× cell suspension. A 2× assay reagent solution of 50 mM Tris pH 7.4, 50 mM KCl, 100 uM EDTA, 0.3× BugBuster (Invitrogen), 20 uM DEVD-Rhodamine-110 (Bachem), 50 kU/mL Lysozyme was also made. Both the 2× cell suspension and the 2× assay reagent were loaded into 1 mL luer lock syringes, which were purged of air and fitted with luer-to-poly ethyl ethyl ketone (PEEK) tubing adapters. The cell syringe used PEEK tubing with 0.005” internal diameter, and all other syringes used 0.015” internal diameter PEEK tubing.

Droplets containing expressed Caspase library variants were generated at the co-flow drop maker junction. Both the 2× cell suspension and the 2× assay reagents flowed into the device at 15 uL/h, and were pinched into droplets by fluorinated oil (HFE 7500) containing 1% (wt/wt) PEG–perfluoropolyether amphiphilic block copolymer surfactant flowing at 100 uL/h.

After incubating on-chip for ~3 min, droplets were sorted using electrocoalescense with an aqueous stream of 10 mM Tris, pH 8, 1 mM EDTA. A 473 nm laser was focused onto the channel just upstream of the sorting junction, each droplet was individually excited, and its fluorescence emission measured using a spectrally filtered PMT at 520 nm. A field-programmable gate array card controlled by custom LabVIEW code analyzed the droplet signal at 200 kHz, and if it detected sufficient fluorescence, a train of eight 180 V, 40 kHz pulses was applied by a high-voltage amplifier. This pulse destabilized the interface between the droplet and the adjacent aqueous stream, causing the droplet to merge with the stream via a thin-film instability, after which the droplet contents were injected into the collection stream via its surface tension. The contents of the sorted droplets were collected in a microcentrifuge tube for further processing. Droplets were processed at 800–1000 Hz. Because the cell occupancy of the droplets was 10%, we analyzed 80–100 cells per second. Caspase-3 and −7 libraries were sorted in triplicate over a total of 6 days. In total we analyzed.

### DNA recovery and sequencing

Recovered plasmid DNA was purified using Zymo spin columns and transformed into ultra-high efficiency 10 G Supreme *E. coli* cells (Lucigen). Cells cultured in SOC media and recovered for 45 min at 37 °C. The recovered culture was then used in totality to inoculate a larger 200 mL culture which was incubated overnight until its OD_600_ reached 0.5. The larger cultures were pelleted and resuspended in 20 mL 25% glycerol for storage at −80 °C. Dilutions of the culture were plated prior to incubation to measure how many transformants were present. We generally observed 0.75 –1x as many transformants as what we sorted. Plasmid purified from the larger culture was digested with the restriction enzyme DraIII and ScoI, gel extracted, tagmented using the Nextera XT Library Prepration Kit (Illumina) and sequenced using the Illumina MySeq 2 × 300. Read coverage for the sequencing runs are displayed in Supplementary Fig. 4. Reads with a quality score <30 were discarded.

### DMS data processing and analysis

The reads from the Illumina FASTQ files were mapped to the caspase reference gene using Bowtie2 [48], and translated to amino acid sequences. Mutations observed fewer than ten times were discarded prior to continuing analysis. Fitness effects of each observed amino acid substitution was estimated using a positive-unlabeled learning framework that compares sequences from the presorted population with the sorted population [39, 49]. Full sequencing databases can be found in the National Center for Bioinformatics Sequence Read Archive (NCBI SRA) under the following accession codes: SRX8049113, SRX8049114, SRX8049115, SRX8049116, SRX8049117, SRX8049118, SRX8049119, SRX8049120, SRX8049121, SRX8049122, SRX8049123, SRX8049124. Python and R scripts used to analyze data can be found at https://github.com/RomeroLab/pudms and https://github.com/RomeroLab/DMS-analysis.

## Supplementary information

Supplemental material

## Data Availability

The datasets generated during and/or analyzed during the current study are available in the National Center for Bioinformatics Sequence Read Archive (NCBI SRA) under the following accession codes: SRX8049113, SRX8049114, SRX8049115, SRX8049116, SRX8049117, SRX8049118, SRX8049119, SRX8049120, SRX8049121, SRX8049122, SRX8049123, SRX8049124.
