# Correction to: Microfluidic deep mutational scanning of the human executioner caspases reveals differences in structure and regulation

**DOI:** 10.1038/s41420-022-00873-1

**Published:** 2022-02-18

**Authors:** Hridindu Roychowdhury, Philip A. Romero

**Affiliations:** 1 Department of Biochemistry, University of Wisconsin-Madison, Madison, WI USA; 2 Department of Chemical & Biological Engineering, University of Wisconsin-Madison, Madison, WI USA; 3 The University of Wisconsin Carbone Cancer Center, Madison, WI USA

**Keywords:** Proteases, Enzyme mechanisms, Biotechnology, Screening

Correction to: *Cell Death Discovery* 10.1038/s41420-021-00799-0, published online 10 January 2022

The original version of this article unfortunately contained an error in an author name. Hridindu Roychowdhury was incorrectly spelled as “Roychowdury”. The correct spelling is “Roychowdhury”. The original article has been corrected.

