# Supplementary material for: Microfluidic deep mutational scanning of the human executioner caspases reveals differences in structure and regulation
Source: Cell Death Discov. 2022 Jan 10;8:7. doi: 10.1038/s41420-021-00799-0 (PMC8748541; doi:10.1038/s41420-021-00799-0)
Supplement: Supplementary file 1 — Supplemental material [file 41420_2021_799_MOESM1_ESM.docx]

**Supplemental Information**

**Supplemental Table 1:** **Caspase Screening Statistics** CASP3 and CASP7 were sorted in triplicate. Each sort lasted for 6.5-8 hours and we recovered at least 10^5^ variants per sort.

|  | Replicate | Drops analyzed | Positive drops sorted | Time (hrs) | Analysis frequency (Hz) | Sorting frequency (Hz) | Fraction of drops sorted | CFU recovered | Fraction functional caspases after sorting |
| --- | --- | --- | --- | --- | --- | --- | --- | --- | --- |
| CASP3 | 1 | 15,639,854 | 693,057 | 7.3 | 592 | 26.2 | 4% | 450,000 | 80% |
|  | 2 | 6,886,651 | 420,630 | 6.6 | 291 | 17.8 | 6% | 200,000 | 70% |
|  | 3 | 15,061,487 | 499,529 | 7 | 598 | 19.8 | 3% | 100,000 | 66% |
| CASP7 | 1 | 19,202,100 | 593,495 | 8 | 670 | 20.7 | 3% | 280,000 | 85% |
|  | 2 | 22,474,041 | 602,890 | 8.3 | 755 | 20.3 | 3% | 480,000 | 90% |
|  | 3 | 17,601,832 | 414,992 | 7 | 698 | 16.5 | 2% | 120,000 | 90% |

**Supplemental Table 2:** Recombinant CASP mutants expressed in *E. coli* were assayed for proteolytic activity against the fluorescent substrate DEVD-Rhodamine-110 at a range of concentrations. Enzyme concentration was determined by active site titration using the irreversible pan-caspase inhibitor Z-VAD-FMK. Measurements were taken in triplicate and uncertainty calculated as the standard error.

| **Enzyme** | **[Enzyme] (nM)** | ***V_max_* (nMol/s)** | ***K_m_* (uM)** | ***K_cat_* (s^-1^)** | ***K_cat_* / *K_m_***  **(s^-1^uM^-1^)** |
| --- | --- | --- | --- | --- | --- |
| **WT CASP3** | 200 +/- 20 | 2.78 +/- 0.06 | 21.0 +/- 0.9 | 0.13 +/- 0.007 | 0.70 +/- 0.06 |
| **CASP3 D175A** | 190 +/- 100 | n.m. | n.m. | n.m | n.m. |
| **CASP3 G177R** | 170 +/- 20 | 2.29 +/- 0.04 | 8.2 +/- 0.3 | 0.28 +/- 0.01 | 1.60 +/- 0.05 |
| **WT CASP7** | 80 +/- 20 | 1.33 +/- 0.04 | 45 +/- 2 | 0.03 +/- 0.002 | 0.40 +/- 0.01 |
| **CASP7 H144A** | 180 +/- 70 | n.m. | n.m. | n.m | n.m. |
| **CASP7 Q196A** | 150 +/- 20 | 0.94 +/- 0.04 | 61 +/- 4 | 0.02 +/- 0.001 | 0.10 +/- 0.02 |
| **CASP7 D198A** | 100 +/- 20 | 0.92 +/- 0.05 | 68 +/- 3 | 0.0054 +/- 0.0003 | 0.05 +/- 0.02 |
| **CASP7 F241G** | 80 +/- 20 | 0.92 +/- 0.05 | 44 +/- 4 | 0.13 +/- 0.02 | 0.25 +/- .05 |


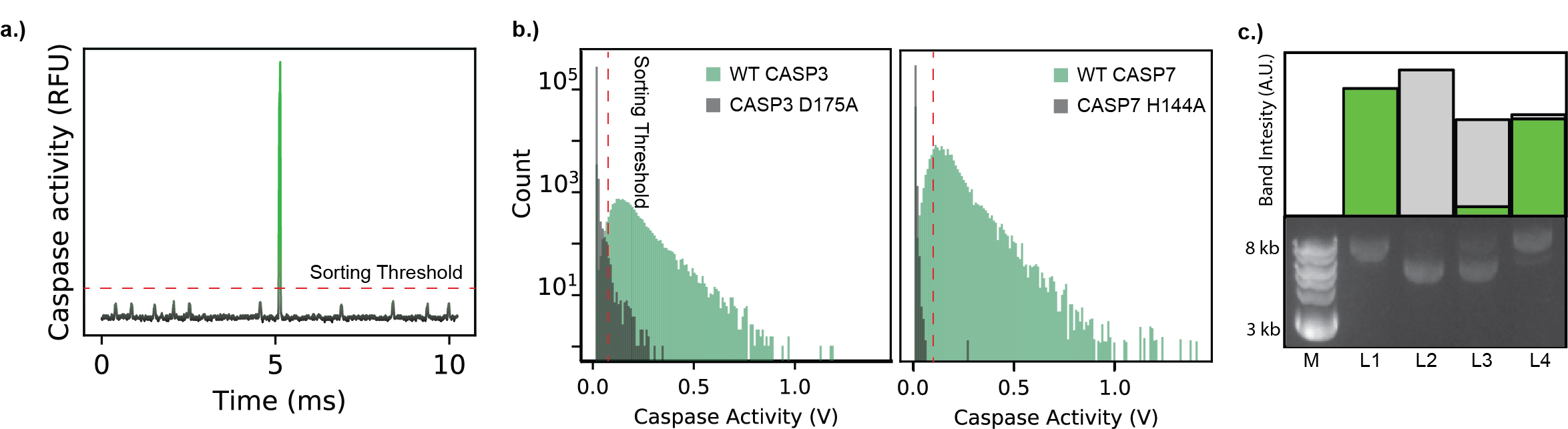


**Supplemental Figure 1:** (a) A time trace of droplets in the microfluidic device as they cross the fluorescence detector. The small peaks correspond to inactive or empty droplets, while the large peak in the center is a droplet containing active caspases. (b) A histogram of fluorescence activity for WT CASP3, WT CASP7 and their respective inactive variants, CASP3 D175A and CASP7 H144A as they are observed in the microfluidic device. WT CASP3 and CASP7 display significantly higher fluorescence signal than CASP3 D175A or CASP7 H144A droplets. (c) Quantification of recovered plasmid from a mock sorting experiment containing a 10:1 mixture of empty pET22 plasmid and CASP3-containing plasmid. Lanes M: NEB 1kb+ DNA standard; L1: pET 22 plasmid containing WT CASP3; L2: pET 22 plasmid with no insert; L3: a 10:1 mixture of empty vector to CASP3 plasmid containing *E. coli* cells; L4: plasmid recovered after microfluidic screening the L3 input showing significant enrichment of CASP3 plasmid.

**
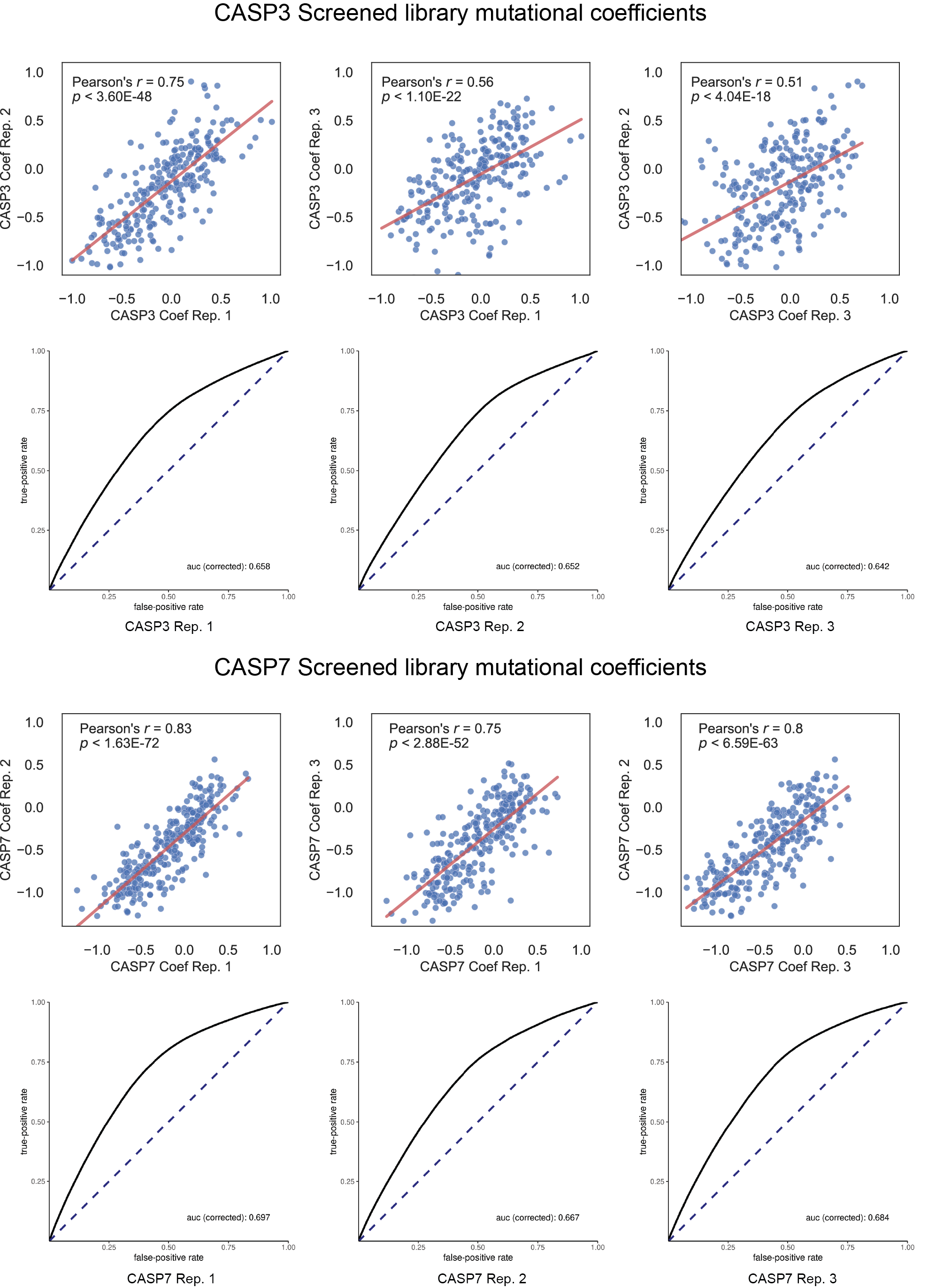
**

**Supplemental Figure 2: Correlation of regression coefficients across experimental replicates.** For each experimental replicate, the regression coefficient at each site is plotted against each other. We have also included the receiver operating characteristic (ROC) curves for the PU learning on each experimental replicate. All three CASP7 experimental replicates correlate well with each other. CASP3 replicates 1 and 2 correlate well with each other, however replicate 3 correlates poorly with the others and was not used for further analysis. It’s likely the microfluidic sorting in replicate 3 had sorting errors that resulted in false positive sequences.

**
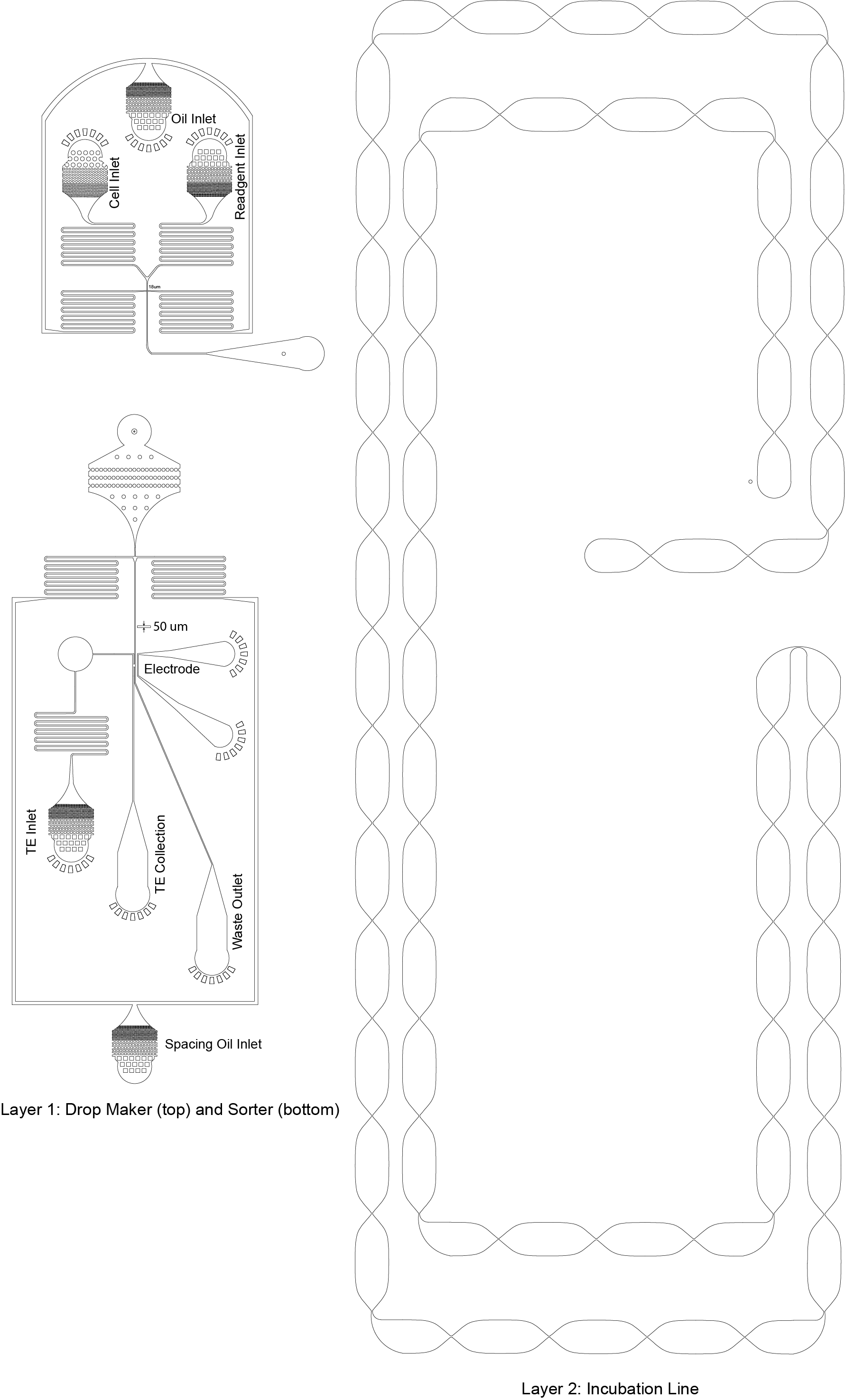
**

**Supplemental Figure 3: Scale schematic of the microfluidic design used in this study.** Layer 1 features the 15 um-tall drop-maker and sorter. All inlets are labeled as such. Layer 2 is the 50 um-tall incubation line that connects the drop maker and the sorter. The “sausage-like” repeating pattern serves to randomize the position of droplets transverse to the direction of flow to average out the effects of laminar flow that makes droplets in the center move faster than droplets near the edge of the channel.


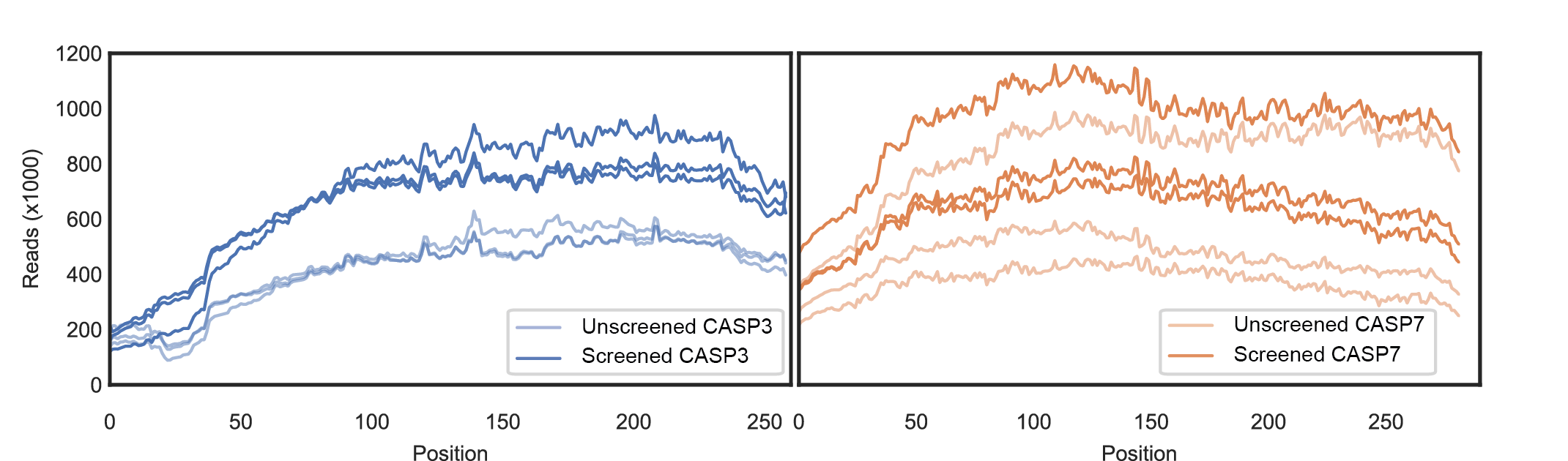


**Supplemental Figure 4: Read coverage of Illumina Sequencing runs for CASP3 and CASP7 datasets.**

Each sequenced library (Unscreened CASP3, Screened CASP3, Unscreened CASP7, Screened CASP7) displays at least 200k reads at each position. Mutations that appeared fewer than 10 times at any given position were assumed to be noise and not considered in downstream analysis.
